# Vortioxetine Improves Context Discrimination in Mice Through a Neurogenesis Independent Mechanism

**DOI:** 10.3389/fphar.2018.00204

**Published:** 2018-03-12

**Authors:** Daniela Felice, Jean-Philippe Guilloux, Alan Pehrson, Yan Li, Indira Mendez-David, Alain M. Gardier, Connie Sanchez, Denis J. David

**Affiliations:** ^1^Université Paris-Saclay, Univ. Paris-Sud, Faculté de Pharmacie, CESP, INSERM UMRS1178, Chatenay-Malabry, France; ^2^Lundbeck Research USA, Paramus, NJ, United States; ^3^Translational Neuropsychiatry Unit, Department of Clinical Medicine, Aarhus University, Risskov, Denmark

**Keywords:** vortioxetine, context discrimination, neurogenesis, c-Fos, mice

## Abstract

Major Depressive Disorders (MDD) patients may exhibit cognitive deficits and it is currently unclear to which degree treatment with antidepressants may affect cognitive function. Preclinical and clinical observations showed that vortioxetine (VORT, an antidepressant with multimodal activity), presents beneficial effects on aspects of cognitive function. In addition, VORT treatment increases adult hippocampal neurogenesis (AHN) in rodents, a candidate mechanism for antidepressant activity. Pattern separation (PS) is the ability to discriminate between two similar contexts/events generating two distinct and non-overlapping representations. Impaired PS may lead to overgeneralization and anxiety disorders. If PS impairments were described in depressed patients, the consequences of antidepressant treatment on context discrimination (CD) are still in its infancy. We hypothesized that VORT-increased AHN may improve CD. Thus, in an attempt to elucidate the molecular mechanism underpinning VORT treatment effects on CD, a rodent model of PS, the role of AHN and stress-induced c-Fos activation was evaluated in the adult mouse hippocampus. Chronic treatment with VORT (1.8 g/kg of food weight; corresponding to a daily dose of 10 mg/kg, 3 weeks) improved CD in mice. Interestingly, chronic treatment with VORT reversed ablation of AHN-induced delay in CD and freezing behavior. VORT treatment decreased stress-induced c-Fos activation in the dorsal but not ventral dentate gyrus. VORT treatment did not affect c-Fos activity in the hippocampus of mice with ablated neurogenesis. This study highlights a role of VORT in CD, which may be independent from AHN and hippocampal c-Fos activation. Further studies elucidating the mechanisms underlying VORT’s effects in CD could contribute to future strategies for alleviating the disease burden for individuals suffering from depression and/or anxiety disorders.

## Introduction

Major depressive disorders (MDD) and anxiety disorders are common health problems in today’s society and display high comorbidity although the pathologies differ. MDD patients may also exhibit cognitive dysfunction in various domains, e.g., memory (verbal and visual), learning and attention (Austin et al., 2001; Darcet et al., 2016). MDD may be associated with impairments in pattern separation (PS), a mechanism for encoding very similar memories in non-overlapping and distinct representations. Impairments in PS may lead to overgeneralization of memory, which may contribute to the development of disorders such as post-traumatic stress and panic disorder (Kheirbek et al., 2012). Preclinical evidence suggests that adult hippocampal neurogenesis (AHN) plays a role in context discrimination (CD), a rodent model of PS and that increased AHN improves CD in mice (Sahay et al., 2011a; Guilloux et al., 2017).

It is currently unclear how treatment with antidepressants may affect cognitive functions (Fava, 2003). Although many studies found that a wide range of antidepressant treatments were able to improve both mood and cognitive-related symptoms in MDD (for review Darcet et al., 2016) others failed to show an association between antidepressant treatment and improvement in cognitive functions in depressed patients (Marcourakis et al., 1993; Knegtering et al., 1994; Amado-Boccara et al., 1995; Stein and Strickland, 1998; de Carvalho et al., 2002; Gorenstein et al., 2006; Frampton, 2016). About 50–60% of MDD patients have recurrent episodes (Darcet et al., 2016). This group of MDD patients is the one most affected by cognitive symptoms.

Vortioxetine (VORT) is an antidepressant with multimodal-activity (Tritschler et al., 2014), which combines serotonin (5-HT) transporter inhibition with actions at 5-HT receptors (agonist at 5-HT_1A_; partial agonist at 5-HT_1B_; antagonist at 5-HT_1D_, 5-HT_3_, and 5-HT_7_ receptors) (Sanchez et al., 2015). Recent clinical and preclinical data suggest that treatment with VORT has beneficial effect on aspects of cognitive function (Al-Sukhni et al., 2015; Mahableshwarkar et al., 2015; Sanchez et al., 2015; McIntyre et al., 2016). Specifically, preclinical data demonstrate that VORT improves memory in rats through 5-HT receptor-dependent mechanisms (Mork et al., 2013; du Jardin et al., 2014; Jensen et al., 2014; Betry et al., 2015; Westrich et al., 2015). Similarly, clinical trials suggest that VORT does not affect cognitive functions negatively in healthy subjects (Theunissen et al., 2013) and displays some beneficial effects on cognition in adults and elderly depressed patients (Katona et al., 2012; McIntyre et al., 2014; Mahableshwarkar et al., 2015, 2016; Smith et al., 2017). In addition, chronic treatment with VORT has been shown to increase AHN in 129SvEV mice (Guilloux et al., 2013).

Recent studies have implicated adult-born hippocampal neurons in CD, a process by which similar experiences or events are transformed into discrete, non-overlapping representations (Treves et al., 2008). Cognitive deficits consistent with impaired CD in MDD patients have been also described (Shelton and Kirwan, 2013). Actually, depressed individuals have lower PS abilities than non-depressed individuals. We hypothesized that VORT-increased AHN (Guilloux et al., 2013) may improve CD.

Thus, this study aimed to investigate whether chronic treatment with VORT improved CD in mice. Then, in order to gain further insights in the CD processes targeted by VORT, we investigated whether AHN affects CD response in VORT-treated animals and if hippocampal neuronal activity changes were observed.

## Materials and Methods

### Animals

#### Vortioxetine and Context Discrimination

**FIGURE 1:**
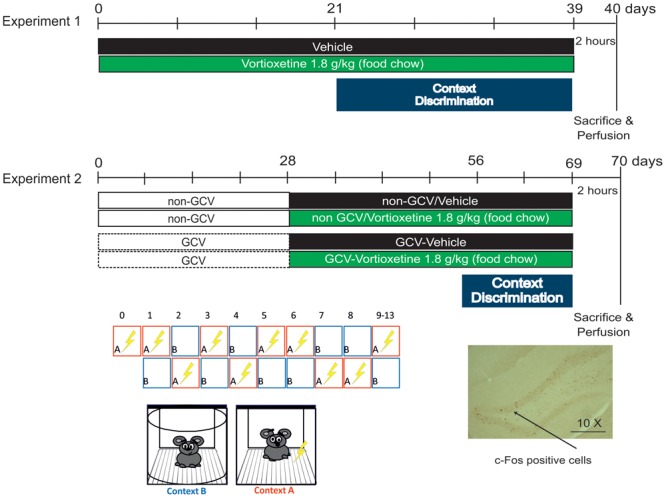
Experimental protocol.

C57BL/6J Rj male mice, 2 months old (Janvier Labs, France) were used to assess CD (see timeline, **Figure 1**).

#### Role of Adult Hippocampal Neurogenesis in Vortioxetine-Increased Context Discrimination

##### GFAP-TK^+^ mouse model

In order to assess the role of AHN on CD, glial fibrillary acidic protein (GFAP)-Thymidine kinase (TK^+^) male mice were used. Mice were generated as previously described (Mendez-David et al., 2017). An agreement (license L-O 15-2015/0) between the NIH and the Université Paris-Sud provides CESP/UMRS 1178 laboratory with the use of transgenic mice that express herpes simplex virus (HSV) TK under control of the GFAP promoter (GFAP-TK mice), as previously described (Snyder et al., 2011; Mendez-David et al., 2017) and developed in the laboratory of Dr. Heather Cameron of the National Institute of Mental Health (NIMH).

Mice were maintained under standard conditions (12/12h light/dark cycle, lights on at 6 AM, 22 ± 1°C, food and water ad libitum, five mice per cage). All behavioral testing was performed during the light cycle from 9:00 to 15:00. The protocols involving animals and their care were conducted in conformity with the institutional guidelines that are in compliance with national and international laws and policies (Council directive # 87-848, October 19, 1987, Ministére de l’Agriculture et de la Forêt, Service Vétérinaire de la Santé et de la Protection Animale, permissions # 92-256B to DD and with the European Communities Council Directive 2010/63/UE) and in compliance with protocols approved by the Institutional Animal Care and Use Committee (CEE26 authorization 2012-099).

### Drug Treatment

#### Vortioxetine (VORT) Treatment

Vortioxetine HBr was incorporated into Purina 5001 rodent chow at a concentration of 1.8 g/kg of food weight (Research Diets, Inc., New Brunswick, NJ, United States). This dose results in engagement of all VORT’s primary pharmacological targets. VORT (1.8 g/kg of food weight; ∼10 mg/kg) was administered for 3 weeks’ in C57BL/6J Rj and 4 weeks’ in GFAP-TK^+^ mice study prior the start of the CD protocol (**Figure 1**). Chronic treatment with VORT has been shown to not affect body weight (**Supplementary Figure S1**).

#### Ganciclovir Administration

Ganciclovir [Rodent Diet, Grain-Based, Valganciclovir (VGCV, Roche, Indianapolis, IN, United states) 165 mg/kg; Custom Animal Diets, LLC] was administered in the chow for 4 weeks prior the start of CD protocol using the method reported in previous studies (Saxe et al., 2006, 2007; Lugert et al., 2017). GCV treatment continued until the end of the study. GCV was weekly removed on Friday, Saturday, and Sunday as described in Lugert et al. (2017) and Mendez-David et al. (2017). GFAP-TK-positive (GFAP-TK^+^) mice received regular chow (NON-GCV) or VGCV, the L-valyl ester of ganciclovir, has a high (approximately 85%) bioavailability and is rapidly converted into ganciclovir by intestinal and hepatic esterases after oral administration. After phosphorylation by HSV-TK ganciclovir is toxic to proliferating cells in S-phase of mitosis.

### Behavioral Analysis

#### Context Discrimination (CD)

This paradigm tests the animals’ ability to distinguish between two similar contexts, conditions that are most likely to recruit the dentate gyrus (DG) (Sahay et al., 2011a). Testing was conducted in chambers (25 cm × 25 cm × 25 cm) with 3 methacrylate walls, 1 aluminum wall, and a stainless steel grid floor, which is encased in a sound attenuating box [67 (W) cm × 53 (D) cm × 55 (H) cm]. The shock associated training context A and the similar (no-shock) context B were characterized by similar features (stainless grid floor, cube box) and non-similar features (context A: rectangular box shape, light on, anise scent; context B: round box shape, light off, lemon scent). For discrimination learning, mice were exposed to the training context in which they received a single 2 s foot shock of 0.4 mA, 185 s following placement in the chamber. Mice were taken out of the chamber 15 s following termination of the foot shock and returned to their home cage. Three hours later, mice were placed in the similar context in which they were left for 180 s and were never shocked. In this study context A (shock-paired) and context B (shock-unpaired) was presented randomly for 8 days, except on day zero in which mice were exposed to context A alone (**Figure 1**). From day 9 of PS, context A was always followed by context B until the end of the test. Measurement of freezing levels in both training context (3 min pre-shock) and in the similar context (3 min) each day allowed assessment of discrimination between the two contexts and was computed as a Discrimination ratio: (Freezing Training context - Freezing similar context)/(Freezing Training context + Freezing similar context). A score of 0 indicates complete lack of discrimination, i.e., freezing levels are the same in the similar and training contexts (Freezing similar context = Freezing Training context), whereas a score of 1 indicates perfect discrimination, i.e., freezing-level in the similar context is zero (Freezing similar context = 0). Behavior was recorded by a digital video camera and freezing behavior was analyzed with Freezing Software version 2.0.04 (Packwin, Harvard apparatus, Bioseb, France).

### c-Fos Immunohistochemistry

Two hours following foot shock stress in context A (**Figure 1**), deeply anesthetized animals (100 mg/ml ketamine; 20 mg/ml xylazine) were perfused transcardially with phosphate buffer solution (PBS) followed by cold paraformaldehyde (PFA, 4% w/v in PBS). Brains were removed and kept in 4% PFA overnight at 4°C. After the post-fixation period, brains were cryo-protected in a 30% sucrose solution to prevent formation of ice crystals. The tissues were snap-frozen in cold isopentane and stored at -80°C. Brains were sliced into 35 μm-thick coronal sections using a Leica CM1900 cryostat. Sections were serially collected in a cryoprotectant solution (25% 0.2M PBS, 30% ethylene glycol, 25% glycerol, 20% H_2_O) and stored at -80°C until use.

c-Fos immunohistochemistry [Experiment 1 (C57BL/6J Rj mice): vehicle (VEH), *n* = 7; VORT, *n* = 7; Experiment 2 (GFAP-TK^+^ mice): NON-GCV/VEH, *n* = 8; GCV/VEH, *n* = 8; NON-GCV/VORT, *n* = 8; GCV/VORT, *n* = 8] was conducted as previously described (O’Mahony et al., 2010). Sections were washed 3 × 5 min in 0.01 M PBS (pH 7.4), and incubated in freshly prepared 0.75% H_2_O_2_ for 20 min to inhibit endogenous peroxidase. Sections were incubated at room temperature for 20 min in 10% Normal Goat Serum, 0.1% Triton-X 100, in PBS to prevent non-specific binding. Sections were then incubated overnight at room temperature with rabbit anti-c-Fos polyclonal antibody (1:5000; Santa Cruz Biotechnology, Santa Cruz, CA, United States) in 0.01 M PBS with 1% v/v NGS. Sections were washed and incubated for 90 min at room temperature with a secondary biotinylated anti-rabbit antibody (1:100, VECSTAIN^®^ Elite^®^ ABC Kit, Vector Laboratories) followed by incubation in ABC reagent (VECSTAIN^®^ Elite^®^ ABC Kit, Vector Laboratories). c-Fos positive cells were detected by incubation with 3, 3-diaminobenzidine tetrahydrochloride (DAB: 0.02% w/v; Sigma-Aldrich, St. Louis, MO, United States) with 0.0075% H_2_O_2_ in PBS. Sections were visualized using an Olympus BX51 microscope; images were captured by an Olympus DP71 digital camera with Cell F software (Olympus Corporation, Tokyo, Japan) and c-Fos positive cells were counted. Coordinates of coronal plates and limits of the structures analyzed were defined according to a mouse brain atlas [(Paxinos and Franklin, 2001); the dorsal hippocampus was defined as anterior–posterior (AP) -0.94 to -2.30 and the ventral hippocampus as AP -2.46 to -3.80]. For each region, three consecutive sections were analyzed and an investigator blind to the experimental conditions counted c-Fos positive cells bilaterally.

### Statistical Analysis

Results are presented as mean ± SEM. Data were analyzed using unpaired two-tailed student’s *t*tests or One/Two-way analysis of variance (ANOVA) repeated measures. Significant main effects or interactions were followed up with Fisher’s least significant difference (LSD) *post hoc* test as appropriate. For all comparisons, *p* < 0.05 was the criterion used for statistical significance. Statistical analyses were performed using GraphPad Prism 6.0h software (GraphPad Software Inc., La Jolla, CA, United States). Statistical outliers were removed from the analysis [outlier = average ± (2^∗^*SD*); Experiment 2: GCV VEH: 2 outliers; VORT: 1 outlier; GCV VORT: 3 outliers]. **Figure 4C**, outliers: 1 outlier in VORT group (**Figure 4C**); 2 outliers in VEH group and 3 outliers in VORT group (**Figure 4D**). DCX analysis, **Supplementary Figure S2**: 2 outliers in VEH group and 1 outlier in VORT group.

## Results

Detailed statistical results are provided in **Supplementary Tables S1–S5**.

### Effects of Chronic Treatment With VORT in CD in Adult C57BL/6J Rj Mice

**FIGURE 2:**
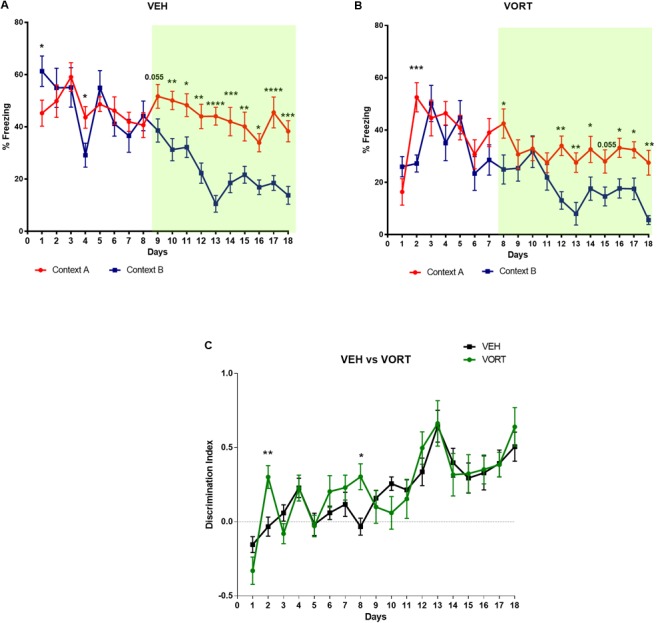
Effects of VORT (1.8 g/kg; ∼10 mg/kg) treatment on CD in C57BL/6J Rj mice. Two-way repeated measures ANOVA of context and day followed by Fisher’s predicted least-square difference *post hoc* tests; ^∗^*p* < 0.05; ^∗∗^*p* < 0.01; ^∗∗∗^*p* < 0.001; ^∗∗∗∗^*p* < 0.0001 (**A,B**: context A versus context B; **C**: VEH versus VORT). The *n* number for CD protocol was: vehicle (VEH), *n* = 10; Vortioxetine (VORT), *n* = 10.

The effects of chronic VORT treatment on CD are shown in **Figure 2** and **Supplementary Tables S1, S2**. Analysis of freezing behavior over days in both contexts (A and B for aversive and non-aversive respectively) revealed a decrease in freezing behavior in context B in VEH-treated group (**Figure 2A**) at day 9 (*p* = 0.055) and started significantly discriminating at day 10 (**Figure 2A** and **Supplementary Table S1**). VORT-treated mice (**Figure 2B**) started freezing less in the non-aversive chamber and started discriminating significantly between the two contexts at day 6 and until the end of the protocol (**Figure 2** and **Supplementary Table S1**). Finally, discrimination index analysis revealed that VORT treated mice discriminate faster than VEH treated mice from day 8 (**Figure 2C** and **Supplementary Table S2**). Overall, this data suggest that VORT improves CD.

### Chronic VORT Treatment Improve Context Discrimination Independently From Adult Hippocampal Neurogenesis

#### Effects of Ablation of Neurogenesis on CD in Adult Mice

**FIGURE 3:**
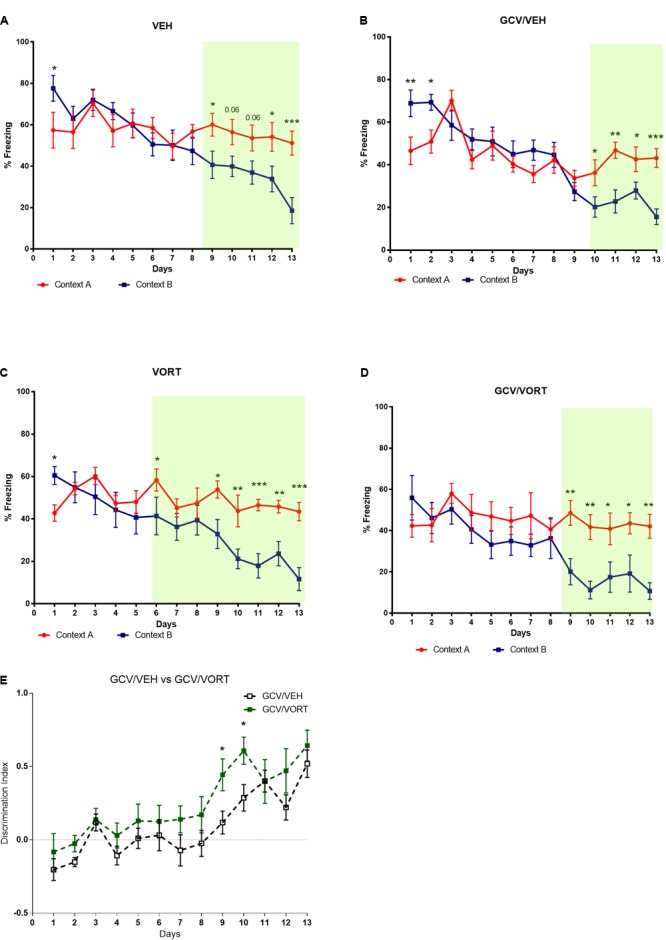
Effects of ablation of adult hippocampal neurogenesis on context discrimination in VEH and VORT (1.8 g/kg; ∼10 mg/kg) treated GFAP-TK TG mice. Two-way repeated measures ANOVA of context and day followed by Fisher’s predicted least-square difference *post hoc* tests; ^∗^*p* < 0.05; ^∗∗^*p* < 0.01; ^∗∗∗^*p* < 0.001; ^∗∗∗∗^*p* < 0.0001 (**A–D**: context A versus context B; **E**: GCV/VEH versus GCV/VORT). The *n* number for CD protocol was: NON-GCV/VEH, *n* = 10; GCV/VEH, *n* = 9; NON-GCV/VORT, *n* = 8; GCV/VORT, *n* = 7. GFAP-TK TG mice, glial fibrillary acidic protein (GFAP)-Thymidine kinase (TK) engineered male; GCV, Ganciclovir; VEH, vehicle; VORT, Vortioxetine.

The consequences of arresting AHN following chronic GCV treatment in GFAP-TK^+^ mice were evaluated on CD (**Figure 3** and **Supplementary Tables S1, S2**). Unlike control group (NON-GCV/VEH GFAP-TK^+^ mice) which discriminated at day 9, GCV/VEH GFAP-TK^+^ mice started discriminating significantly between contexts A and B at day 10 (*p* < 0.05, **Figures 3A,B** and **Supplementary Table S1**). The GCV/VEH GFAP-TK^+^ group kept discriminating until the end of the protocol (day 11, *p* < 0.01; day 12, *p* < 0.05; day 13, *p* < 0.001). If arresting AHN did not fully impaired CD, it delayed CD performances in adult mice.

#### Effects of Vortioxetine Treatment on CD in Mice With Ablated Neurogenesis

Unlike control mice treated with VORT (NON-GCV/VORT GFAP-TK^+^ mice) which discriminated at day 6 (*p* < 0.05) and from day 9 to the end of the protocol (**Figure 3C**), GCV/VORT GFAP-TK^+^ mice started discriminating from day 9 (*p* < 0.01) and kept discriminating until the end of the protocol (**Figure 3D** and **Supplementary Table S1**). It suggests that ablation of neurogenesis delayed CD performances in adult VORT-treated GFAP-TK^+^ mice. However, discrimination index analysis revealed that GCV/VORT GFAP-TK^+^ mice discriminated better than GCV/VEH GFAP-TK^+^ mice (**Figure 3E** and **Supplementary Table S2**; day 9: *p* < 0.05; day 10: *p* < 0.05), indicating that VORT improved CD performances in adult mice.

### Chronic VORT Treatment Decreased Freezing Behavior in C57BL/6J Rj and Reversed the Effects of Arrested AHN on Freezing Behavior in GFAP-TK^+^ Mice

**FIGURE 4:**
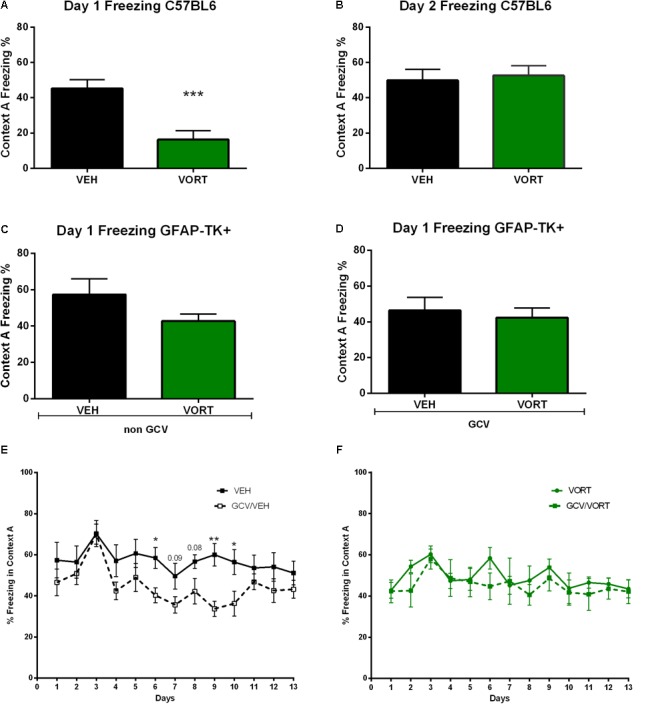
Effects of VORT treatment on freezing behavior in VEH and VORT (1.8 g/kg; ∼10 mg/kg) treated mice. Unpaired two-tailed student’s *t*-tests between VEH and VORT group or Two-way repeated measures ANOVA of context and day followed by Fisher’s predicted least-square difference *post hoc* tests; ^∗^*p* < 0.05; ^∗∗^*p* < 0.01; ^∗∗∗^*p* < 0.001 (**A–D**: VEH versus VORT; **E**: VEH versus GCV/VEH; **F**: VORT versus GCV/VORT). GFAP-TK TG mice, glial fibrillary acidic protein (GFAP)-Thymidine kinase (TK) engineered male; GCV, Ganciclovir; VEH, vehicle; VORT, Vortioxetine.

The effects of VORT treatment on freezing behavior are shown in **Figure 4**. On day 1 of CD protocol, VORT treated mice froze less than VEH treated mice (**Figure 4A**; *t* = 4.072 *df* = 18; *p* < 0.001). However, on day 2 of CD protocol, no difference was detected between the groups (**Figure 4B**; *t* = 0.3243 *df* = 18; *p* = 0,7495).

Unpaired *t*-test analysis revealed no difference in freezing behavior between VEH and VORT group in GFAP-TK^+^ GCV-untreated mice (**Figure 4C**; *t* = 1.419 *df* = 16, *p* = 0, 1750) and GFAP-TK^+^ GCV-treated mice (**Figure 4D**; *t* = 0.4411 *df* = 13; *p* = 0, 6664). However, GCV/VEH GFAP-TK^+^ mice displayed a decrease in freezing behavior in aversive context A when compared to NON-GCV/VEH GFAP-TK^+^ mice (**Figure 4E** and **Supplementary Table S3**), suggesting that arresting AHN decreases freezing behavior *per se*. However, freezing behavior in VORT-treated mice treated/untreated with GCV was unchanged (**Figure 4F** and **Supplementary Table S3**), suggesting that VORT treatment reversed the effects of loss of neurogenesis on freezing activity.

In an attempt to understand the molecular mechanisms underpinning VORT’s effects on CD, shocks-induced c-Fos activation analysis in the hippocampus in C57BL/6J Rj and GFAP-TK^+^ mice were performed.

### Vortioxetine Effects on Shock-Induced c-Fos Activation in the Adult Hippocampus in C57BL/6J Rj Mice

**FIGURE 5:**
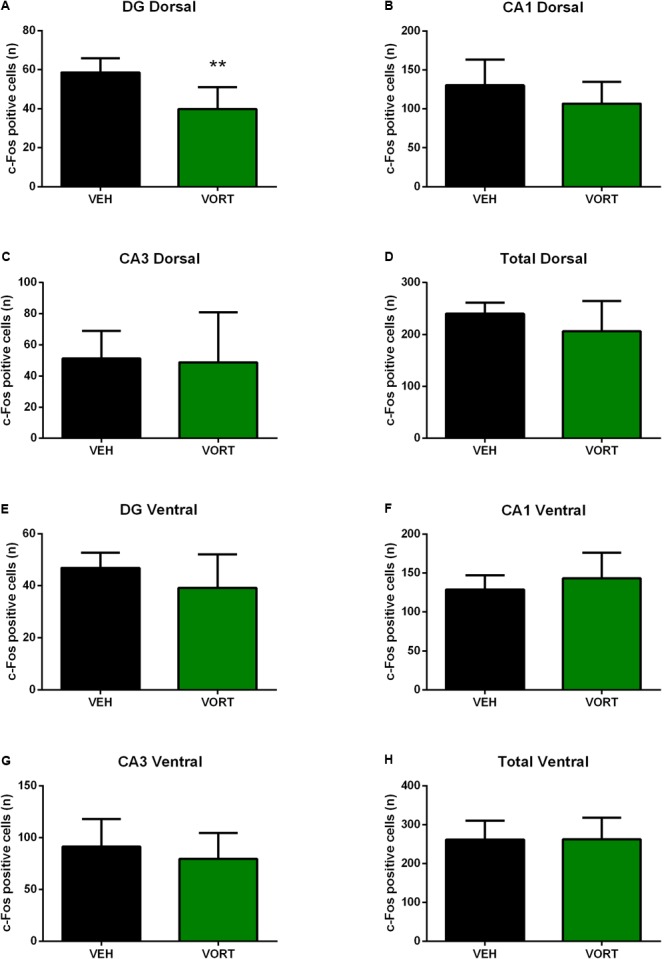
Effects of VORT (1.8 g/kg; ∼10 mg/kg) treatment on shock-induced c-Fos activation in the adult hippocampus in C57BL/6J Rj mice (Dorsal hippocampus, **A–D**; Ventral hippocampus, **E–H**). Unpaired two-tailed student’s *t*-tests between VEH and VORT group; ^∗∗^*p* < 0.01. VEH, vehicle; VORT, Vortioxetine.

The effects of VORT treatment on shock-induced c-Fos activation in the hippocampus are shown in **Figure 5** and statistical analysis is summarized in **Supplementary Table S4**. No effects of chronic VORT treatment were observed in the total ventral hippocampus (**Figure 5H**) and neither in the ventral dentate gyrus (DG; **Figure 5E**), CA1 (**Figure 5F**), or CA3 (**Figure 5G**) areas. VORT treatment did not alter the number of c-Fos positive cells in the dorsal CA1 (**Figure 5B**), dorsal CA3 (**Figure 5C**) areas, and in the total dorsal hippocampus (**Figure 5D**). Unlike the ventral DG, VORT treatment decreased the number of c-Fos positive cells in the dorsal DG when compared to the VEH group (**Figure 5A**; *t* = 3.712 *df* = 12; *p* < 0.01).

### Vortioxetine Treatment Did Not Alter the Number of c-Fos Positive Cells in the Hippocampus of GFAP-TK Mice

**FIGURE 6:**
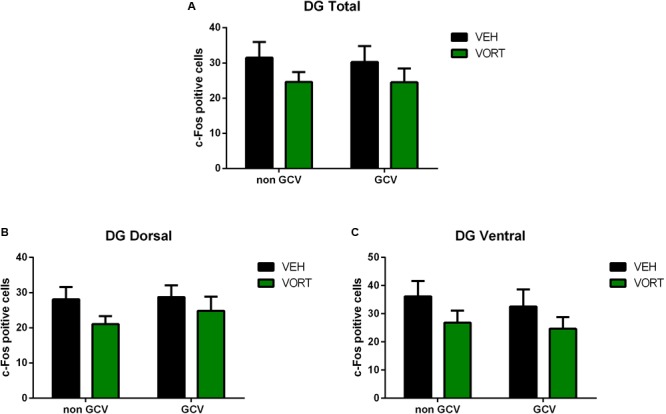
Effects of VORT (1.8 g/kg; ∼10 mg/kg) treatment on shock-induced c-Fos activation in the adult hippocampus (Total DG, **A**; Dorsal DG, **B**; Ventral DG, **C**) in GFAP-TK TG mice. GFAP-TK TG mice, glial fibrillary acidic protein (GFAP)-Thymidine kinase (TK) engineered male; GCV, Ganciclovir; VEH, vehicle; VORT, Vortioxetine.

The effects of VORT treatment on stress-induced c-Fos activation in the hippocampus in GFAP-TK mice are shown in **Figure 6** and statistical values are summarized in **Supplementary Table S5**. Two-way ANOVA analysis (treatment and neurogenesis ablation) revealed that chronic treatment with VORT did not alter the number of stress-induced c-Fos positive cells in the whole DG (**Figure 6A**), dorsal DG (**Figure 6B**), or ventral DG (**Figure 6C**).

## Discussion

In this study, we assessed the effects of chronic treatment with VORT, an antidepressant with multimodal activity, on CD in adult C57BL/6J Rj mice. Importantly in our study the CD protocol was randomized for the first 8 days. Thus time was not an additional cue and mice were not able to predict whether they would be exposed first to the safe (shock non-associated) or aversive (shock-associated) context. Chronic treatment with VORT improved CD in adult mice. VORT-treated mice started discriminating earlier between the two contexts compared to control mice (**Figure 2C**).

It is interesting that VORT treatment is able to improve CD in C57BL/6J Rj mice, which are characterized by a normal cognitive profile. Indeed, C57BL/6J Rj mouse strain displays no cognitive deficits as assessed in different cognitive tasks including a visual discrimination task and a water maze task when compared to other mice strains including 129S2/Sv, BALB/c, C3H/He, CBA/Ca, and DBA/2 mice strains (Brooks et al., 2005). Of note, VORT-treated mice start discriminating between the safe and non-safe context before the CD protocol was switched from randomized to non-randomized in contrast to the control group. We may have detected lager differences between the two groups if the protocol was randomized until the end of the experiment.

In line with our finding, acute VORT administration increased freezing behavior in the retention phase in a contextual fear condition test suggesting increased contextual memory in Sprague–Dawley rats (Mork et al., 2013). In addition, other preclinical findings strongly support a role of VORT in inducing pro-cognitive effects in rodents (Sanchez et al., 2015). For example, treatment with VORT improved episodic short-term memory and spatial memory in rats (novel object recognition, NOR, and Y-maze spontaneous alterations) respectively (Mork et al., 2013; du Jardin et al., 2014). Interestingly, VORT restored the memory impairment in NOR and spontaneous alterations induced by serotonin depletion in rats (Jensen et al., 2014). Similarly, VORT treatment restored reversal learning impaired by 5-HT depletion in rats as assessed by the attentional set-shifting test, a paradigm that measures cognitive flexibility (Wallace et al., 2014; Popik and Nikiforuk, 2015). In parallel, clinical studies supports a role of VORT treatment in cognitive function (Wallace et al., 2014; Smith et al., 2017) and a recent study showed that VORT treatment is able to target directly the neural circuitry implicated in the cognitive deficits in depression (Smith et al., 2017).

The potential mechanisms underpinning VORT effects on CD were also investigated. Recent studies suggested a role of adult neurogenesis in mouse models of PS, namely the ability to discriminate between similar events. Indeed, increased AHN is enough to improve separations behavior in mice (Sahay et al., 2011a). Similarly, ablation of hippocampal neurogenesis (hippocampal x-ray irradiation) has been shown to impair spatial PS in adult mice (Clelland et al., 2009). Moreover, another study showed that AHN is necessary for spatial PS in mouse models (Tronel et al., 2012). Furthermore, mice with genetic ablation of neurogenesis were impaired in spatial PS (Tronel et al., 2012). In addition, AHN may be an adaptive mechanism to optimally encode contextual or olfactory information (Sahay et al., 2011b).

Unlike control NON GCV/VEH GFAP-TK^+^ mice that discriminated from day 9 of the CD protocol (**Figure 3**), GCV/VEH GFAP-TK^+^ mice (ablated neurogenesis) discriminated from day 10 and keep discriminating until the end of the protocol. Thus, if arresting AHN with a genetic ablation strategy did not induce a deficit in CD, it affects negatively the process. Of note, it is important to highlight that administration of GCV for 4 weeks to ablate neurogenesis may have induced compensatory effects or lead to different coping strategies during the CD test. GCV was administered during CD protocol until the end of the experiment as described in (Mendez-David et al., 2017), thus preventing the resume of AHN (Sakalem et al., 2017). As mentioned above several studies support a role of adult neurogenesis in rodent models of PS, however other studies do not find a clear link (Becker, 2016). Recent data suggest that newly generated neurons are highly active and fire continuously over time generating increased pattern overlap and exhibiting less spatial tuning than their mature counterparts (Becker, 2016; Danielson et al., 2016). However, increased AHN may increase CD indirectly by increasing feedback inhibition onto mature granule cells or enhancing pattern integration (Becker, 2016). Intriguingly, AHN decreases with age whereas PS increases with age (Yassa and Stark, 2011). Furthermore, while AHN may play a physiological role in CD, AHN is not required for the pharmacological effects of some drugs on behavior in mice (Gardier et al., 2009).

Knowing that chronic VORT treatment increases AHN (Guilloux et al., 2013), we then investigated whether AHN is required for VORT improved-CD. Surprisingly, arresting AHN did not prevent VORT improved-CD. Indeed, GCV/VORT GFAP-TK^+^ mice started discriminating on day 9 (*p* < 0.01) and kept discriminating until the end of the protocol. Therefore, GFAP-TK^+^ mice treated with VORT started discriminating 1 day earlier than the vehicle group with ablated neurogenesis. In line with previous observations, VORT treatment improved CD. Overall, discrimination index analysis revealed that GFAP-TK^+^ VORT-treated mice discriminated better than the respective VEH group (**Figure 3**, day 10: *p* < 0.05; day 11: *p* < 0.05). In addition, chronic treatment with vortioxetine induced a trend (*p* = 0.06) for increased total number of doublecortin positive cells (DCX), a marker of AHN (Wang et al., 2008) in C57BL/6J Rj mice as observed in recently published study in another mouse strain (Guilloux et al., 2013) (Supplementary Methods and Results and **Supplementary Figure S1**). Overall, our data indicated that VORT improved CD through neurogenesis-independent mechanism. Importantly, only some behavioral paradigms in rodents are neurogenesis dependent while others are neurogenesis independent (Gardier et al., 2009). Indeed, other brain areas different from the hippocampus are clearly involved in modulating rodent behavior such as amygdala and cingulate cortex (Gardier et al., 2009).

Interestingly, VORT-treated mice exhibited significantly lower freezing levels than VEH mice on the first day of the protocol following exposure to shock on day 0 (**Figure 4**). However, VORT-treated mice displayed similar freezing levels in the following days of CD protocol. This finding may suggest either that VORT-treated mice do not recall the shock received on day 0 or that they are less anxious than VEH treated mice. Given VORT-treated mice discriminated earlier than VEH control mice and freezing levels are similar to the VEH group on the following days, most likely VORT induced anxiolytic-like effects. Accordingly, preclinical findings have shown that treatment with VORT induced anxiolytic-like effects in mice (Guilloux et al., 2013) and rats (Mork et al., 2012). Freezing analysis on day 1 revealed no difference in freezing behavior between VEH and VORT groups with or without ablation of neurogenesis in GFAP-TK^+^ mice. This contradicting finding may be explained by different genetic makeup. Intriguingly, freezing behavior analysis revealed that GCV/VEH GFAP-TK^+^ (ablated neurogenesis) display decreased freezing behavior when compared to the NON-GCV/VEH GFAP-TK^+^ group, whereas mice treated with VORT displayed similar freezing behavior when neurogenesis is ablated (**Figure 4**). Thus, VORT treatment prevented reduction in freezing behavior, i.e., generalization.

In an attempt to further elucidate the mechanisms underpinning VORT effects on CD we investigated shock-induced c-Fos activation in the hippocampus (**Figures 5, 6**). Importantly, recent studies suggest that the hippocampus does not work as one unit but dorsal and ventral hippocampus may have different functions. Thus, the dorsal hippocampus is more involved in mediating cognitive functions, whereas the ventral hippocampus is more important in the control of stress responses (Bannerman et al., 1999, 2004). Intriguingly, VORT decreased c-Fos activation in the dorsal but not ventral dentate gyrus in the hippocampus. No main effect was observed in other areas of the hippocampus including CA1, CA3, or the total hippocampus. Surprisingly, no differences in stress-induced c-Fos activation were observed between groups in the dorsal and ventral hippocampus in GFAP-TK^+^ mice. Similar to the freezing behavior analysis, this may be due to genetic background differences. Indeed, different mice strain may display different c-Fos brain activation in response to the same stress (O’Mahony et al., 2010). Similarly, different rat strains exhibit a differential expression of c-fos mRNA in response to restraint stress (Trneckova et al., 2006). Our data highlight a role of the dorsal dentate gyrus in CD, however the meaning of decreased c-Fos expression in the dorsal dentate gyrus is unclear. In agreement with a role of the dorsal hippocampus in CD, a study showed that ablation of the dorsal but not ventral hippocampus impairs CD in mice delaying acquisition (Wu and Hen, 2014). Interestingly, this finding was observed only when the two contexts were presented to mice not randomly (Wu and Hen, 2014). In a randomized protocol both dorsal and ventral hippocampal play a role (Wu and Hen, 2014). Our data may indicate that although VORT modulates hippocampal neuronal activity this effect is not necessary to observe the behavioral effects on CD as observed in GFAP-TK^+^ mice.

## Conclusion

According to our data, mice with ablated neurogenesis are still able to acquire the CD protocol and are able to discriminate between two similar contexts (one safe and one aversive). Thus, AHN is not necessary to acquire CD. However, mice with ablated neurogenesis exhibited a slight impairment of learning the protocol, i.e., 1 day later compared to control mice. This may suggest that AHN is not necessary but may play a role since the acquisition of CD protocol is delayed. However, VORT effects on CD are independent of AHN since no behavioral difference was observed when neurogenesis was ablated. Furthermore, the dorsal but not ventral DG may play a role in VORT effects on CD as revealed by c-Fos analysis in C57BL6 Rj mice. In parallel, c-Fos analysis in the hippocampus revealed no difference in the DG and CA1 and CA3 hippocampal areas activation following an electric shock in mice with ablated neurogenesis and the respective control group. This data suggest that activation of the adult hippocampus may not be responsible for the behavioral phenotype observed in the CD. These data could be expected, since the role and contribution of the DG and AHN to CD is still unclear and a matter of debate (Yassa and Stark, 2011).

Overall, the findings further support a role of VORT in cognition. Future studies should investigate the effects of VORT treatment on CD in animal models of depression such as chronic mild stress or social defeat. These models may give more insights than this study conducted in naïve mice. An ongoing clinical trial (NCT02969876) investigates the effects of VORT in PS and its potential mechanisms of action in depressed patients. Further elucidating the mechanisms underlying VORT effects in CD could contribute to future strategies for alleviating the disease burden for individuals suffering from depression and/or anxiety disorders.

## Author Contributions

DF, DD, AG, J-PG, AP, YL, and CS contributed to the conceptualization and design of study and writing of the manuscript. DF conducted the experiments. IM-D contributed to the experimental work. DF, DD, AG, and J-PG performed the data analysis.

## Conflict of Interest Statement

AP, YL, and CS were fulltime employees at Lundbeck when the studies were conducted. DD serves as a consultant for Lundbeck, Roche, and Servier. AG receives research fundings from Servier. The other authors declare that the research was conducted in the absence of any commercial or financial relationships that could be construed as a potential conflict of interest.

## Supplementary Material

FIGURE S1 Effects of VORT (1.8 g/kg; ∼10 mg/kg) treatment on body weight in C57BL/6J Rj mice.

FIGURE S2 Effects of VORT (1.8 g/kg; ∼10 mg/kg) treatment on doublecortin (DCX) immunostaining in the adult dentate gyrus of the hippocampus of C57BL/6J Rj mice. Unpaired one-tailed student’s *t*-tests between VEH and VORT group; Abbreviations: VEH, vehicle; VORT, Vortioxetine.

TABLE S1 Statistical results for context discrimination paradigm (**Figures 2A,B, 3A–D**).

TABLE S2 Statistical results for discrimination index analysis (**Figures 2C, 3E**).

TABLE S3 Statistical results for freezing behavior in context A of CD paradigm (**Figures 4E,F**).

TABLE S4 Statistical results for the effects of VORT treatment on shock-induced c-Fos activation in the adult hippocampus in C57BL/6J Rj mice (**Figure 5**).

TABLE S5 Statistical results for the effects of VORT treatment on shock-induced c-Fos activation in the adult hippocampus in GFAP-TK TG mice (**Figure 6**).
