# Supplementary figures and images for: Vortioxetine Improves Context Discrimination in Mice Through a Neurogenesis Independent Mechanism
Source: Front Pharmacol. 2018 Mar 12;9:204. doi: 10.3389/fphar.2018.00204 (PMC5857583; doi:10.3389/fphar.2018.00204)

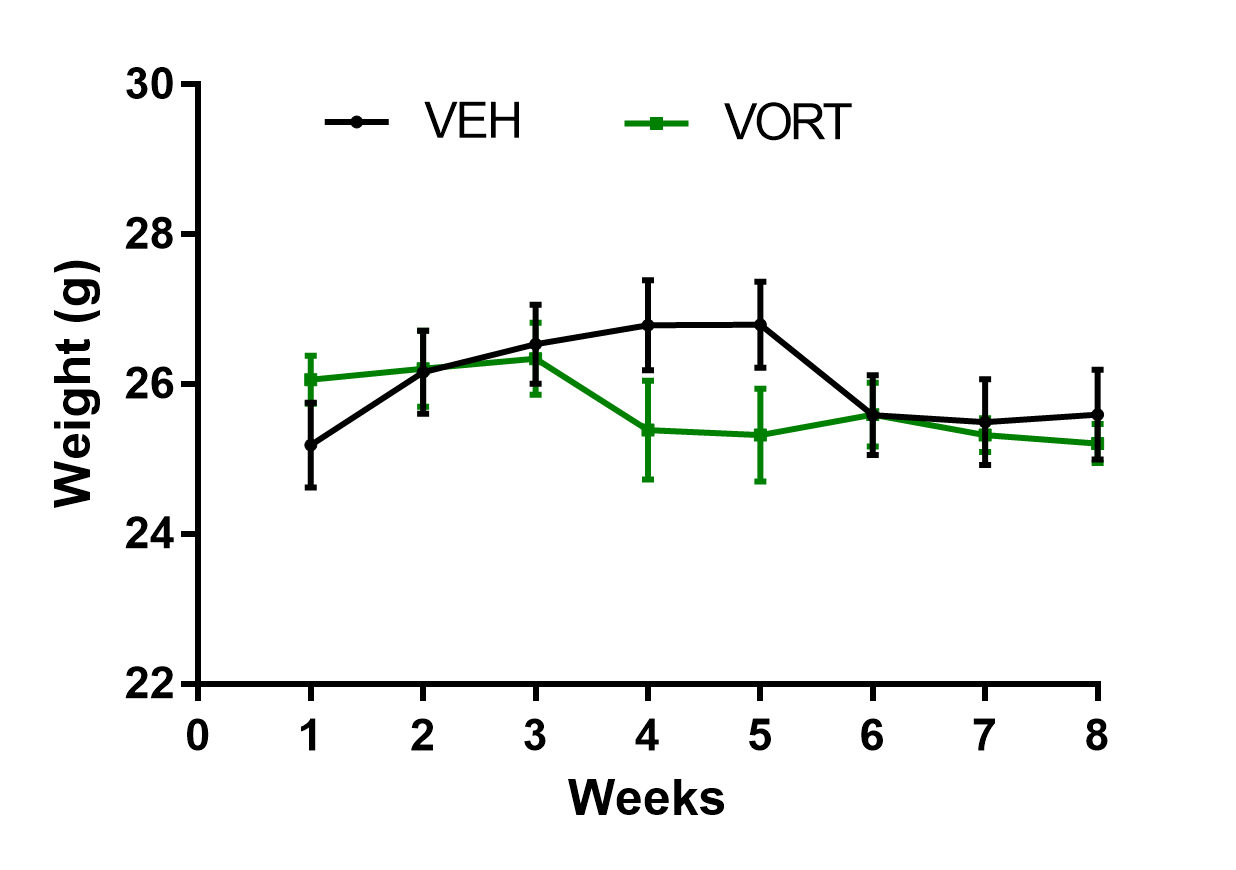

Supplement: FIGURE S1 — Effects of VORT (1.8 g/kg; ∼10 mg/kg) treatment on body weight in C57BL/6J Rj mice. [file Image_1.TIF]

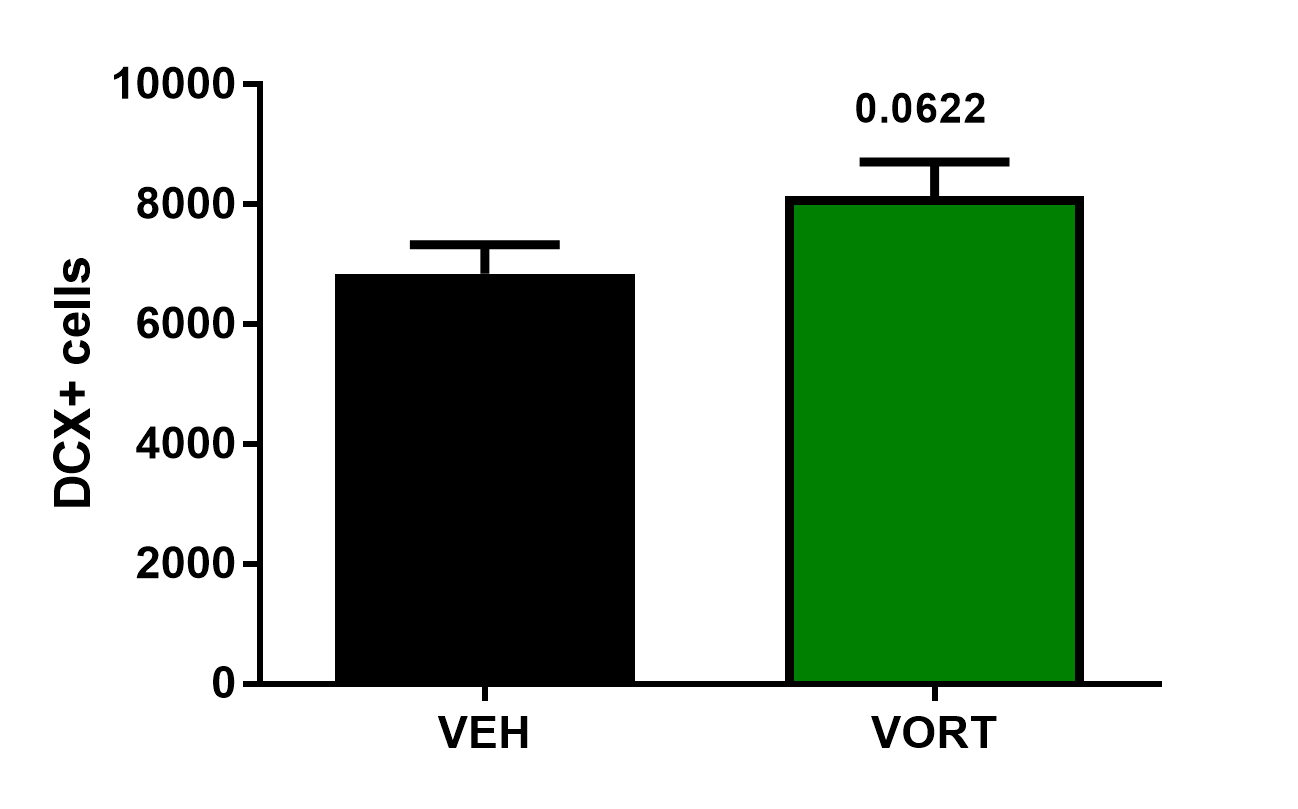

Supplement: FIGURE S2 — Effects of VORT (1.8 g/kg; ∼10 mg/kg) treatment on doublecortin (DCX) immunostaining in the adult dentate gyrus of the hippocampus of C57BL/6J Rj mice. Unpaired one-tailed student’s t-tests between VEH and VORT group; Abbreviations: VEH, vehicle; VORT, Vortioxetine. [file Image_2.TIF]
