# Supplementary material for: Vortioxetine Improves Context Discrimination in Mice Through a Neurogenesis Independent Mechanism
Source: Front Pharmacol. 2018 Mar 12;9:204. doi: 10.3389/fphar.2018.00204 (PMC5857583; doi:10.3389/fphar.2018.00204)
Supplement: TABLE S1 — Statistical results for context discrimination paradigm (Figures 2A,B, 3A–D). [file Table_1.DOCX]

Supplementary Table 1

| Figure | Parameter | Treatment group numbers | Total number of samples | Two-Way ANOVA F-value Context | Two-Way ANOVA P-value Context | Treatment group numbers | Total number of samples | Two-Way ANOVA F-Value Time | Two-way ANOVA P-value Time | Treatment group numbers | Total number of samples | Two-way ANOVA F-value Interaction | Two-way ANOVA P-value Interaction | Day 1: Context A vs Context B | Day 2: Context A vs Context B | Day 3: Context A vs Context B | Day 4: Context A vs Context B | Day 5: Context A vs Context B | Day 6: Context A vs Context B | Day 7: Context A vs Context B | Day 8: Context A vs Context B | Day 9: Context A vs Context B | Day 10: Context A vs Context B | Day 11: Context A vs Context B | Day 12: Context A vs Context B | Day 13: Context A vs Context B | Day 14: Context A vs Context B | Day 15: Context A vs Context B | Day 16: Context A vs Context B | Day 17: Context A vs Context B | Day 18: Context A vs Context B |
| --- | --- | --- | --- | --- | --- | --- | --- | --- | --- | --- | --- | --- | --- | --- | --- | --- | --- | --- | --- | --- | --- | --- | --- | --- | --- | --- | --- | --- | --- | --- | --- |
| 2A | % Freezing | 1 | 18 | 10.57 | <0.01 | 17 | 306 | 11.22 | <0.0001 | 17 | 306 | 5.068 | <0.0001 | - | - | - | - | - | - | - | - | 0.055 | ** | * | ** | **** | *** | ** | * | **** | *** |
| 2B |  | 1 | 18 | 8.043 | <0.05 | 17 | 306 | 8.51 | <0.0001 | 17 | 306 | 2.671 | <0.0001 | - | *** | - | - | - | - | - | * | - | - | - | ** | ** | * | 0.055 | * | * | ** |
| 3A |  | 1 | 18 | 1.223 | 0.2834 | 12 | 216 | 8.841 | <0.0001 | 12 | 216 | 4.596 | <0.0001 | - | - | - | - | - | - | - | - | * | 0.06 | 0.06 | * | *** |  |  |  |  |  |
| 3B |  | 1 | 16 | 0.2579 | 0.2579 | 12 | 192 | 15.08 | <0.0001 | 12 | 192 | 6.595 | <0.0001 | - | - | - | - | - | - | - | - | - | * | ** | * | *** |  |  |  |  |  |
| 3C |  | 1 | 14 | 4.018 | 0.0648 | 12 | 168 | 9.998 | <0.0001 | 12 | 168 | 5.623 | <0.0001 | * | - | - | - | - | * | - | - | * | ** | *** | ** | *** |  |  |  |  |  |
| 3D |  | 1 | 12 | 4.632 | 0.0524 | 12 | 144 | 4.384 | 0.0001 | 12 | 144 | 2.775 | 0.0001 | - | - | - | - | - | - | - | - | ** | ** | * | * | ** |  |  |  |  |  |
