# Supplementary material for: Vortioxetine Improves Context Discrimination in Mice Through a Neurogenesis Independent Mechanism
Source: Front Pharmacol. 2018 Mar 12;9:204. doi: 10.3389/fphar.2018.00204 (PMC5857583; doi:10.3389/fphar.2018.00204)
Supplement: TABLE S2 — Statistical results for discrimination index analysis (Figures 2C, 3E). [file Table_2.DOCX]

Supplementary Table 2

| Figures | Parameter | Treatment group numbers | Total number of samples | Two-Way ANOVA F-value Treatment | Two-Way ANOVA P-value Treatment | Treatment group numbers | Total number of samples | Two-Way ANOVA F-Value Time | Two-way ANOVA P-value Time | Treatment group numbers | Total number of samples | Two-way ANOVA F-value Interaction | Two-way ANOVA P-value Interaction | Day 1: Vehicle vs Vortioxetine | Day 2: Vehicle vs Vortioxetine | Day 3: Vehicle vs Vortioxetine | Day 4: Vehicle vs Vortioxetine | Day 5: Vehicle vs Vortioxetine | Day 6: Vehicle vs Vortioxetine | Day 7: Vehicle vs Vortioxetine | Day 8: Vehicle vs Vortioxetine | Day 9: Vehicle vs Vortioxetine | Day 10: Vehicle vs Vortioxetine | Day 11: Vehicle vs Vortioxetine | Day 12: Vehicle vs Vortioxetine | Day 13: Vehicle vs Vortioxetine | Day 14: Vehicle vs Vortioxetine | Day 15: Vehicle vs Vortioxetine | Day 16: Vehicle vs Vortioxetine | Day 17: Vehicle vs Vortioxetine | Day 18: Vehicle vs Vortioxetine |
| --- | --- | --- | --- | --- | --- | --- | --- | --- | --- | --- | --- | --- | --- | --- | --- | --- | --- | --- | --- | --- | --- | --- | --- | --- | --- | --- | --- | --- | --- | --- | --- |
| 2C | Discrimination Index | 1 | 18 | 0.3136 | p=0.5824 | 17 | 306 | 11.95 | p<0.0001 | 17 | 306 | 1.484 | 0.0988 | ** | - | - | - | - | - | - | * | - | - | - | - | - | - | - | - | - | - |
| 3E |  | 1 | 18 | 5.365 | p<0.05 | 12 | 180 | 13.61 | p<0.0001 | 12 | 180 | 13.61 | p=0.7581 | - | - | - | - | - | - | - | - | * | * | - | - | - |  |  |  |  |  |
