# Supplementary material for: Vortioxetine Improves Context Discrimination in Mice Through a Neurogenesis Independent Mechanism
Source: Front Pharmacol. 2018 Mar 12;9:204. doi: 10.3389/fphar.2018.00204 (PMC5857583; doi:10.3389/fphar.2018.00204)
Supplement: TABLE S3 — Statistical results for freezing behavior in context A of CD paradigm (Figures 4E,F). [file Table_3.DOCX]

Supplementary Table 3

| Figure | Parameter | Treatment group numbers | Total number of samples | Two-Way ANOVA F-value Treatment | Two-Way ANOVA P-value Treatment | Treatment group numbers | Total number of samples | Two-Way ANOVA F-Value Time | Two-way ANOVA P-value Time | Treatment group numbers | Total number of samples | Two-way ANOVA F-value Interaction | Two-way ANOVA P-value Interaction | Day 1: Vehicle/Vortioxetine vs GCV group | Day 1: Vehicle/Vortioxetine vs GCV group | Day 1: Vehicle/Vortioxetine vs GCV group | Day 1: Vehicle/Vortioxetine vs GCV group | Day 1: Vehicle/Vortioxetine vs GCV group | Day 1: Vehicle/Vortioxetine vs GCV group | Day 1: Vehicle/Vortioxetine vs GCV group | Day 1: Vehicle/Vortioxetine vs GCV group | Day 1: Vehicle/Vortioxetine vs GCV group | Day 1: Vehicle/Vortioxetine vs GCV group | Day 1: Vehicle/Vortioxetine vs GCV group | Day 1: Vehicle/Vortioxetine vs GCV group | Day 1: Vehicle/Vortioxetine vs GCV group |
| --- | --- | --- | --- | --- | --- | --- | --- | --- | --- | --- | --- | --- | --- | --- | --- | --- | --- | --- | --- | --- | --- | --- | --- | --- | --- | --- |
| 4E | % Freezing in Context A | 1 | 17 | 5.021 | p<0.05 | 12 | 204 | 4.046 | p<0.0001 | 12 | 204 | 1.052 | p=0.4032 | - | - | - | - | - | * | 0.09 | 0.08 | ** | * | - | - | - |
| 4F |  | 1 | 13 | 0.7168 | p=0.4125 | 12 | 156 | 1.759 | 0.0595 | 12 | 156 | 0.436 | 0.9468 | - | - | - | - | - | - | - | - | - | - | - | - | - |
