# Supplementary material for: Vortioxetine Improves Context Discrimination in Mice Through a Neurogenesis Independent Mechanism
Source: Front Pharmacol. 2018 Mar 12;9:204. doi: 10.3389/fphar.2018.00204 (PMC5857583; doi:10.3389/fphar.2018.00204)
Supplement: TABLE S4 — Statistical results for the effects of VORT treatment on shock-induced c-Fos activation in the adult hippocampus in C57BL/6J Rj mice (Figure 5). [file Table_4.DOCX]

Supplementary Table 4

| Brain Region | Unpaired two-tailed student's t-tests values |
| --- | --- |
| Total Ventral Hippocampus | t=0.014; df=12; p= 0.9892 |
| Ventral DG | t=1.428 df=12; p= 0.1787 |
| Ventral CA1 | t=1.005 df=12; p= 0.3346 |
| Ventral CA3 | t=0.856 df=12; p= 0.4090 |
| Total Dorsal Hippocampus | t=1.446 df=11; 0.1759 |
| Dorsal DG | t=3.712 df=12; p<0.01 |
| Dorsal CA1 | t=1.445 df=12; p= 0.1740 |
| Dorsal CA3 | t=0.175 df=12; p= 0.8640 |
