# Supplementary material for: Vortioxetine Improves Context Discrimination in Mice Through a Neurogenesis Independent Mechanism
Source: Front Pharmacol. 2018 Mar 12;9:204. doi: 10.3389/fphar.2018.00204 (PMC5857583; doi:10.3389/fphar.2018.00204)
Supplement: TABLE S5 — Statistical results for the effects of VORT treatment on shock-induced c-Fos activation in the adult hippocampus in GFAP-TK TG mice (Figure 6). [file Table_5.DOCX]

Supplementary Table 5

| Brain Region | Two way ANOVA analysis |
| --- | --- |
| Whole DG | Treatment, F(1,28)= 2.527, p= 0.1231; neurogenesis ablation, F(1,28)= 0.02570, p= 0.8738; Interaction: F(1.28)= 0.02175; p= 0.8838 |
| Dorsal DG | Treatment, F(1,28)= 2.647, p= 0.1149; neurogenesis ablation, F(1,28)= 0.4308, p= 0.5169; Interaction: F(1.28)= 0.2077; p= 0.6521 |
| Ventral DG | Treatment, F(1,28)= 2.930, p= 0.0980, 0.1231; neurogenesis ablation, F(1,28)= 0.3201, p= 0.5761; Interaction: F(1.28)= 0.02118; p= 0.8853 |
