# Supplementary material for: Vortioxetine Improves Context Discrimination in Mice Through a Neurogenesis Independent Mechanism
Source: Front Pharmacol. 2018 Mar 12;9:204. doi: 10.3389/fphar.2018.00204 (PMC5857583; doi:10.3389/fphar.2018.00204)
Supplement: Supplementary file 8 [file Data_Sheet_1.DOCX]

Supplementary

**1. Methods**

**1.1 Doublecortin (DCX) Immunohistochemistry**

Doublecortin (DCX) immunohistochemistry was conducted as previously described ([David et al., 2009](#_ENREF_1)) and ([Mendez-David et al., 2014](#_ENREF_2)) and conducted to assess the number of newborn neurons in the hippocampus following chronic treatment with vortioxetine (Vh, n=8; Vortioxetine, n=8). Briefly, sections were rinsed in 0.1 M TBS (3x15 min) and treated with 0.3% H2O2 in 1X PBS/methanol (1:1) for 15 min in order to quench endogenous peroxidase activity and enhance dendritic stain. Sections were incubated in 10% normal donkey serum (NDS) in 0.1 M TBS with 0.5% Triton X-100 for 2 hours, followed by incubation with the goat anti-DCX primary antibody (1: 500; Santa Cruz, #SC 8066) in TBS/Triton X/NDS overnight at 4°C. The secondary antibody used was biotinylated donkey anti-goat (1:500) (Jackson ImmunoResearch, #705-065-003, PA, USA) in TBS for 2 hours at room temperature. DCX immunostaining was revealed by avidin-biotin complex (Vector, CA, USA) and DAB kit.

**2. Results:**

**2.2 The effects of treatment with vortioxetine on DCX positive cells in the adult mouse hippocampus**

The effects of vortioxetine treatment in cell maturation (DCX immunohistochemistry) are shown in Suplementary Figure 1 and statistical. Vortioxetine treatment induced a trend in increasing the number of DCX positive cells when compared to the vehicle group in the dentate gyrus of the hippocampus (unpaired one-tailed student's t-tests analysis: vehicle vs vortioxetine: t=1,663 df=11; p= 0,0622).

**REFERENCES**

David DJ, Samuels BA, Rainer Q, Wang JW, Marsteller D, Mendez I, Drew M, Craig DA, Guiard BP, Guilloux JP, Artymyshyn RP, Gardier AM, Gerald C, Antonijevic IA, Leonardo ED, Hen R (2009) Neurogenesis-dependent and -independent effects of fluoxetine in an animal model of anxiety/depression. Neuron 62:479-493.

Mendez-David I, David DJ, Darcet F, Wu MV, Kerdine-Romer S, Gardier AM, Hen R (2014) Rapid anxiolytic effects of a 5-HT(4) receptor agonist are mediated by a neurogenesis-independent mechanism. Neuropsychopharmacology : official publication of the American College of Neuropsychopharmacology 39:1366-1378.
